# RNAi in Insects: A Revolution in Fundamental Research and Pest Control Applications

**DOI:** 10.3390/insects11070415

**Published:** 2020-07-03

**Authors:** Olivier Christiaens, Jinzhi Niu, Clauvis Nji Tizi Taning

**Affiliations:** 1 Department of Plants and Crops, Ghent University, 9000 Ghent, Belgium; tiziclauvis.taningnji@ugent.be; 2 Key Laboratory of Entomology and Pest Control Engineering, College of Plant Protection, Southwest University, Chongqing 400716, China; jinzhiniu@swu.edu.cn

**Keywords:** RNA interference, crop protection, antiviral immunity, gene silencing, loss-of-function analysis

## Abstract

In this editorial for the Special Issue on ‘RNAi in insect pest control’, three important applications of RNA interference (RNAi) in insects are briefly discussed and linked to the different studies published in this Special Issue. The discovery of the RNAi mechanism revolutionized entomological research, as it presented researchers with a tool to knock down genes, which is easily applicable in a wide range of insect species. Furthermore, RNAi also provides crop protection with a novel and promising pest control mode-of-action. The sequence-dependent nature allows RNAi-based control strategies to be highly species selective and the active molecule, a natural biological molecule known as double-stranded RNA (dsRNA), has a short environmental persistence. However, more research is needed to investigate different cellular and physiological barriers, such as cellular uptake and dsRNA degradation in the digestive system in insects, in order to provide efficient control methods against a wide range of insect pest species. Finally, the RNAi pathway is an important part of the innate antiviral immune defence of insects, and could even lead to applications targeting viruses in beneficial insects such as honeybees in the future.

## 1. Introduction

In 1998, a breakthrough paper from the lab of Andrew Fire and Craig Mello was published in Nature, reporting that the introduction of a target gene-specific double-stranded RNA (dsRNA) into the nematode *Caenorhabditis elegans* led to an efficient gene expression knockdown [1]. Although RNAi-effects had already been observed much earlier in plants, such as fungi and *C. elegans*, this was the first time researchers could prove that dsRNA was the trigger [2]. In the following years, the different RNAi-related pathways were further elucidated in nematodes [3,4,5,6,7,8,9,10]. It also quickly became apparent that these pathways were evolutionarily conserved throughout all eukaryotes and were involved in various processes, including antiviral defence (small interfering RNA or siRNA pathway), internal gene regulation (microRNA or miRNA pathway) and protection of the genome against transposons (piwi-interacting RNA or piRNA pathway) [11,12,13,14]. In insects, these three pathways largely operate through different (but related) effector proteins, although some interaction between them has been suggested [15].

The pathway that was first discovered by Fire and colleagues in *C. elegans* was the siRNA pathway, which is triggered by exogenous double-stranded RNA. In insects, this pathway is very important as part of the innate immune system, offering a first line of defence against viral infections [16]. While this pathway evolved to deal with viral dsRNA in the cell, leading to silencing of crucial viral genes, researchers can exploit this system to introduce exogenous dsRNA, targeting an endogenous gene in the cell. This discovery has led to a revolution in invertebrate molecular biology research, and also in insects, since it allowed for functional genomics loss-of-function studies in non-model species [17]. In 2007, two studies also delivered a proof-of-concept that RNAi could be a promising mode-of-action for pest control products [18,19]. The strategy here would be to effectively silence essential genes in a pest organism, leading to the death of the pest. This approach could offer major benefits from a biosafety point of view, due to its high species-selectivity and the short environmental fate of the active molecule [20].

## 2. RNAi as a Functional Genomics Tool in Insect Research

Before the RNAi breakthrough, most molecular and genetic research in insects was conducted in just a few model insects, such as *Drosophila melanogaster* and *Tribolium castaneum*. The discovery of RNAi allowed researchers to conduct loss-of-function experiments in a much wider range of insect species for which it was much more difficult to obtain stable mutants. Some of the earliest examples include studies in honeybees [21], mosquitoes [22], moths [23] and also non-insect arthropods such as ticks [24]. Over the past twenty years, RNAi protocols have been developed and described for a large number of insect species and a plethora of functional genomics studies have been conducted in non-model insects and have given us insight into the genomic and functional diversity within the insect clade [20].

In the current Special Issue, Ji et al. [25] used RNAi to investigate the role of the so-called imitation switch (ISWI) gene in temperature tolerance in the whitefly *Bemisia tabaci*. The research showed that expression of this gene is inducible by thermal stress and silencing of the gene led to a significantly reduced tolerance to both heat and cold stress. Another study investigated the role of the β-N-acetylglucosaminidase 2 encoding gene (*LsNAG2*) in the development of the cigarette beetle *Lasioderma serricorne* [26]. In 5th instar larvae, knockdown of *LsNAG2* led to significantly reduced expression of genes involved in chitin synthesis and eventually led to abnormal larval-pupal moulting. Injection into pupa eventually led to abnormalities in adult eclosion and wing development.

These studies highlight the value of RNAi as a molecular tool, and while novel gene editing techniques such as CRISPR-Cas9 will also revolutionize research in non-model insects, RNAi will remain an important asset since it allows for transitive knockdown of a gene. Indeed, investigating the function of genes which are crucial for the (early) development or for the survival of an organism might be difficult using gene editing techniques. RNAi, on the other hand, allows researchers to—to some degree—modulate the level of expression and the duration of knockdown by controlling the dose of dsRNA that is administered.

## 3. RNAi-Based Insect Pest Control

Besides the application of RNAi in studying gene functions, its sequence-dependent mode of action has, over the last decade, also drawn much interest to its exploitation for crop protection [27,28]. A growing number of research studies have confirmed that RNAi triggers such as artificial microRNAs (amiRNAs), hairpin-structured RNAs (hpRNAs) and dsRNAs can be specifically designed to selectively target the expression of specific genes or groups of similar gene sequences in a target organism. As such, a target species or a group of species could be targeted while leaving non-target species unharmed [29,30]. This sequence-dependent mode of action, based on a natural molecule as the active ingredient, gives RNAi unique features of efficiency and selectivity compared to other conventional agrochemicals.

For field application, the RNAi approach in the context of insect pest control can be applied in planta, through the production of genetically modified (GM) crops expressing the RNAi trigger against a target species. Several studies have highlighted the potential of this strategy in insect pest control [18,19,31] and recently the first GM maize crop (MON87411), expressing dsRNA against the western corn rootworm, *Diabrotica virgifera virgifera*, has received approval for cultivation in more than 15 countries [32]. However, the GM RNAi-based approach faces several challenges ranging from technical difficulties to transform some crop species, to expensive capital requirements and public acceptance of GM crops. These challenges have triggered research into alternative delivery strategies that mainly rely on the exogenous application of dsRNA against target pests. Examples of such delivery strategies include the spray of formulated dsRNA by itself or via microbes, root drenching, seed soaking and trunk injection (reviewed by Taning et al. [27] and Joga et al. [28]). Exogenously applied dsRNA via plants or directly on the target insect pest has been demonstrated in several studies to induce RNAi-mediated silencing in the target pest.

In the current Special Issue, Lu et al. (2020) reported on the feasibility of significantly reducing the survival of the pest beetle, *Henosepilachna vigintioctopunctata*, following exposure to exogenously applied gene-specific dsRNA. By topically treating detached plant leaves with bacterially expressed dsRNA targeting the *Snf7* gene, which encodes an essential cellular component of endosomal sorting complexes required for transport, they could successfully cause 98%, 88%, and 60% mortality in 1st and 3rd instars, and adults after 10, 12, and 14 days, respectively. Similarly, Ullah et al. (2020) also reported that survival and fecundity was significantly reduced by 41% and 48%, respectively, in the cotton-melon aphid, *Aphis gossypii*, after exposure to dsRNA targeting the *chitin synthase 1* gene.

These studies, among many others, highlight the potential of exploiting RNAi in pest control. However, it should be noted that the choice and effectiveness of the RNAi-mediated delivery strategy will ultimately depend on specific features of the target insect species in question, for example; the effectiveness of the RNAi machinery, host–pathogen interaction mechanisms and structural characteristics. One important barrier affecting RNAi efficacy for pest control is the stability of dsRNA in the environment and in the insect digestive tract [33]. Proof of the impact of digestive nucleases on RNAi efficacy was provided in the current Special Issue by Giesbrecht et al. [34] in the mosquito *Aedes aegypti*. Co-delivery of dsRNA targeting two midgut-specific nucleases with dsRNA targeting a reporter gene led to an increase in silencing efficiency compared to mosquitoes, which were not fed the nuclease-specific dsRNA [34]. One way to address the dsRNA persistence issues could be the application of bacteria producing insect-specific dsRNA, rather than application of naked dsRNA. The bacteria could potentially lead to a longer persistence of the dsRNA in the environment and in the insect. In the current Special Issue, Zhang et al. [35] provide proof that application of dsRNA-expressing bacteria could be used to target the leaf beetle *Plagiodera versicolora*.

## 4. The Role of RNAi in Insect Immunity

Beyond pest control, RNAi can also be applied to protect beneficial insects against viral infections or other pathogens, by aiding their natural immune system. As mentioned earlier, RNAi has evolved naturally as an important first line of defence against viral pathogens in insects. Viral infections generate virus-related dsRNAs, such as replication intermediates, the viral (dsRNA) genome itself, virus-encoded siRNAs or viral transcript–genome hybrids. Those virus-related dsRNAs then trigger the RNAi pathway, leading to a degradation of viral RNAs and eventually the restriction of viral replication and protection of the host. In an attempt to use this natural pathway to improve the antiviral response, several research papers have shown that injecting or even feeding bees with dsRNA that specifically targets viral sequences can lead to lower infection rates [36,37,38]. This could lead to future applications using RNAi to protect beneficial insects from viruses or other pathogens [39].

Further research on the role of RNAi in the innate immune response is also necessary to better understand the cross-talking between RNAi and other immune pathways and to understand the effect of viral or other infections on RNAi efficacy, as this can affect the potential application in pest control. Further optimization and a more efficient RNAi induction by exogenous application of dsRNA is needed for many insects. So far, research has always focused on the siRNA machinery in the search to improve RNAi efficacy. However, from an immunity point of view, the picture is much more complex: (1) the RNAi pathway includes three sub-pathways (siRNA, miRNA and piwiRNA). Application of artificially synthesized dsRNA induces not only expression of the siRNA pathway core genes but also those of the miRNA pathway, suggesting an interactive network of RNAi core genes in aphids [15]. In addition, the crosstalk between RNAi and other non-RNAi immune mechanisms (e.g., JAK/STAT) were also reported, reflecting the sophisticated immunity network of insects [40]. (2) Virus-encoded suppressors can also inhibit the activity of the RNAi pathway. The increased use of next-generation sequencing has led to the detection of novel viruses, especially RNA viruses, among which some have been reported to closely interact with some components of the RNAi machinery. Additionally, insects deal with various viruses. Studies in oriental fruit flies showed that multiple infections of various virus species were detected and RNAi was comprehensively involved in processing the viral dsRNA, thereby producing massive amounts of viral-derived siRNA [41]. Further studies are required to better understand the detailed role of RNAi as a defence system against these newly reported viruses and how these viruses counter the RNAi mechanism. (3) Since the siRNA pathway is first of all a key antiviral pathway in insects, the fitness costs of exploiting this RNAi pathway on a large scale should be considered. Could the exposure to RNAi-based products lead to effects in non-target organisms at the level of the immune system? It has been described that exposure to non-specific dsRNAs leads to a stimulation of the RNAi pathway and different immune pathways in bumblebees, for example [42,43]. However, whether this has any consequences in terms of fitness cost or whether it changes the capacity to fight off viral or other infections has not been investigated so far.

For certain species, the insect–virome interactions vary, which may reflect different situations of RNAi-based immunity in different insect pests. Therefore, further research into the characteristics of RNAi’s natural role in insect immunity is required in various non-model species, including pests and beneficial insects. This information will inform us on the role of the immune system on RNAi efficacy and will help us in optimising the RNAi efficiency in coordination with RNAi’s nature role. And vice versa, these investigations will also offer us more information on the effect of using the RNAi technology on the immune system.

## 5. Conclusions

Here, we described some of the successes that have been achieved in RNAi insect research, the promise RNAi holds in crop protection and the importance of RNAi in the insect antiviral immune system. We also briefly discussed the studies published in the current RNAi Special Issue. While RNAi-based pest control products are expected to come to the market in the next few years, further research into the RNAi mechanism itself and the different barriers affecting RNAi efficacy will be necessary to allow this technology to be used against a wider range of insect species. Furthermore, challenges still lie ahead to assess and minimize the risk of off-target silencing and also to avoid the possibility of resistance emergence to RNAi-based control. It is also worth noting that RNAi-based control strategies are not envisaged as standalone pest control strategies, but should rather be part of a synergistic approach in an integrated pest management (IPM) context, to improve the sustainability of future pest control. Further research implementing this technology in IPM programs is therefore also necessary in the future.
